# Neuronal Apoptosis and Synaptic Density in the Dentate Gyrus of Ischemic Rats’ Response to Chronic Mild Stress and the Effects of Notch Signaling

**DOI:** 10.1371/journal.pone.0042828

**Published:** 2012-08-09

**Authors:** Shaohua Wang, Yang Yuan, Wenqing Xia, Fengfei Li, Yan Huang, Yi Zhou, Yijing Guo

**Affiliations:** 1 Department of Endocrinology, affiliated ZhongDa Hospital of Southeast University, Nanjing, People’s Republic of China; 2 Department of Neurology, affiliated ZhongDa Hospital of Southeast University, Nanjing, People’s Republic of China; Charité-Universitätsmedizin Berlin, Germany

## Abstract

Our previous research highlighted an inconsistency with Notch1 signaling-related compensatory neurogenesis after chronic mild stress (CMS) in rodents suffering from cerebral ischemia, which continue to display post-stroke depressive symptoms. Here, we hypothesize that CMS aggrandized ischemia-related apoptosis injury and worsened synaptic integrity via gamma secretase-meditated Notch1 signaling. Adult rats were exposed to a CMS paradigm after left middle cerebral artery occlusion (MCAO). Open-field and sucrose consumption testing were employed to assess depression-like behavior. Gene expression of pro-apoptotic Bax, anti-apoptotic Bcl-2, and synaptic density-related synaptophysin were measured by western blotting and real-time PCR on Day 28 after MCAO surgery. CMS induced depressive behaviors in ischemic rats, which was accompanied by an elevation in Bax/bcl-2 ratio, TUNEL staining in neurons and reduced synaptophysin expression in the dentate gyrus. These collective effects were reversed by the gamma-secretase inhibitor DAPT (N-[N-(3,5-difluorophenacetyl-L-alanyl)]-S-phenyl-glycine t-butyl ester). We found that post-stroke stressors made neurons in the dentate gyrus vulnerable to apoptosis, which supports a putative role for Notch signaling in neural integrity, potentially in newborn cells’ synaptic deficit with regard to preexisting cells. These findings suggest that post-stroke depression therapeutically benefits from blocking gamma secretase mediated Notch signaling, and whether this signaling pathway could be a therapeutic target needs to be further investigated.

## Introduction

The pathophysiology of post-stroke depression (PSD) remains elusive because of its proposed multifactorial nature. Psychological and social stressors associated with stroke are considered the primary causes of depression [1], [2]. An intriguing property of the adult hippocampus is its potential to generate new neurons throughout life [3], which contributes to functional plasticity [4] under physiological or pathological conditions. Evidence from many studies strongly supports the novel hypothesis that adult hippocampal neurogenesis is reduced in major depression [5]–[7]. Chronic mild stress (CMS) reportedly impairs neurogenesis in the dentate gyrus (DG), which is a proposed etiological factor in depressive disorders and a drug target of antidepressants [8].

Transient global [9] or focal ischemia [10], [11] provokes the migration newly born neurons from the subgranular zone (SGZ) into the granule cell layer of the DG. They subsequently incorporate into the synaptic circuitry. Ischemia-stimulated compensatory neurogenesis can aid rehabilitation after injury and facilitate cognitive recovery. In one of our previous studies, depressive behaviors in ischemic rats were accompanied by reduced DG neurogenesis [12]. Additionally, there is a divergence in the expected activity of the highly conserved Notch-1 (Notch) cell signaling system in the hippocampus during neurogenesis in ischemic animals experiencing chronic mild stress (CMS) [13]. Although neurogenesis and depressive symptoms appear causally connected, some inconsistencies in the relationship between neurogenesis and behavior exist in PSD animals (CMS-treated ischemic stroke animal) relative to controls [12]. Elevated neurogenesis in CMS-treated ischemic stroke animals is accompanied with depressive symptoms. This finding implies that neural integrity is vital for the replacement functions of newborn neurons during ischemic injury. Notch-1 is a plasma membrane receptor that regulates cell fate decisions in the developing nervous system and might govern synaptic plasticity in the adult brain [14]–[16]. Ligand binding (e.g., with Delta and Jagged) results in proteolytic cleavage of Notch. Cleavage in the transmembrane domain is accomplished by the presenilin-1 and gamma-secretase enzyme complex. Subsequently, Notch intracellular domain (NICD) is released and translocates to the nucleus, where it regulates transcription. We postulated that ischemia-stimulated neurogenesis is counterbalanced by the concurrent demise of granule cells via apoptosis, which is another consequence of ischemia [17], [18]. In the hippocampus of ischemic animals experiencing CMS, gamma secretase–mediated Notch signaling is potentially involved in apoptosis and the impaired formation of newborn synapses.

To test this hypothesis, we investigated neuronal apoptosis and synaptic density in the DG of adult ischemic rats in response to chronic mild stress. We examined the preventive effects of Notch signaling inhibition with the gamma secretase inhibitor DAPT. This secretase is required for the release of the Notch intracellular domain, which is stimulated by receptor-ligand interactions. The putative PSD animal model involved cerebral ischemia induced by left middle cerebral artery occlusion followed by exposure to chronic mild stress combined with solitary housing. This preclinical study will advance the general understanding of (1) the potential role of Notch signaling in post-stroke apoptosis that impairs synapse formation between inherent neurons and newborn neurons and (2) neural integrity as a vital characteristic for the replacement functions of DG neurogenesis during ischemic injury. These collective studies will determine if post-stroke stressors are paramount components of the multifactorial pathophysiology of PSD.

## Materials and Methods

### 1. Animals

Adult male Sprague-Dawley rats (210–250 g; Medical College of Southeast University, China) were allowed to acclimate for 2 weeks to their surroundings prior to experimentation. During the two-week period, the animals were trained to consume a 1% (w/v) sucrose solution. Training consisted of three baseline sucrose test sessions (1 hour each) after 20 h of food and water deprivation. Animals were housed 2 per clear plastic cage (40 cm long × 25 cm wide × 15 cm high) with wood-shavings bedding, and given laboratory rat chow and either tap water ad libitum unless otherwise indicated. All rats were kept on a 12-hour light/dark cycle (lights on at 7∶00 a.m.) in the same colony room. Temperature (21±2°C) and humidity (55%) remained constant. All experiments were conducted in compliance with the National Institute of Health’s Guidelines for the Care and Use of Laboratory Animals (NIH Publications No. 80-23; revised in 1996).

### 2. Treatment Regime and Behavioral Tests

On the basis of their baseline sucrose intake, total 42 rats were divided into matched groups as follows: sham operation (CON) group(n = 6), CMS group(n = 6) and surgery group(n = 30). Left MCAO was performed in the surgery group. 18 survivors with a neurological score [19] ≥1 but <4 were randomly divided into ischemic stroke group (MCAO, n = 6)MCAO + CMS group (n = 6) and DPAT-treated (MCAO + CMS + D) groups (n = 6). MCAO + CMS + D group was intraperitoneally injected with DAPT (100 mg/kg) just after post-surgery recovery. CMS group, and then MCAO+CMS group and MCAO + CMS + D group were subjected to CMS with an isolated rearing procedure for 18 days. CON group (Sham-operated animals) underwent the basic surgical procedure, but the thread was not inserted into the common carotid artery. Bcl-2, bax and synaptophysin expression along with neuronal apoptosis in the dentate gyrus were analyzed on day 28 after ischemic surgery. Subsequently, open-field and sucrose preference tests were performed. There were six rats per group.

A detailed description of MCAO plus CMS regimen has been reported in our previous work [20] with minor modifications. Briefly, animals were anaesthetized with sodium pentobarbital (40 mg/kg.) intraperitoneally. Then MCAO was carried out using an intraluminal thread introduced via common carotid artery (CCA) upto the origin of the MCA. Using a heating pad, the animal’s body temperature was continually monitored and maintained at 37°C throughout the surgical procedure and during post-surgery recovery. Neurological evaluation was performed 24 h after the induction of ischemia and scored on a 5-point scale, as proposed by Longa et al (1989), and then CMS regimen began.

The CMS regimen contained 9 different stressors randomly arranged during 18 consecutive days: 20 h food and water deprivation, 18 h water deprivation, 17 h of 45° cage tilt, overnight illumination, 21 h wet cage, 5 min swimming in water at 4°C, 30 min on a 160 Hz rocking bed, 1 min tail pinch and 2 h immobilization. The behavioral tests were employed 28 days after ischemia. In open field testing (OFT), the field consisted of a wooden box (75 cm square chamber, 40 cm high walls) with black walls and a white floor, which was divided by 1-cm-wide black lines into 25 (5×5) equal squares. The OFT was used to evaluate general locomotor and rearing activity of the animals. Locomotor activity was defined as at least three paws in a quadrant and rearing behaviour defined as the animal standing upright on its hind legs were tallied. Both locomotor activity and rearing were manually recorded by trained observers who were blind to the condition of the animals over a 5-min period. Animals were tested individually and only once. For the sucrose preference test, the test subjects were allowed to consume water and 1% sucrose solution for 1 h after 20 h of food and water deprivation. The position of the 2 bottles (left/right sides of the cages) varied randomly between trials. For each trial, the position of both bottles was counterbalanced across the rats in each group. During the test, both bottles were removed after 30 min and weighed. They were replaced by another pair of preweighed bottles (the position of the 2 bottles was reversed). A baseline preference test was performed before the CMS procedure. The sucrose preference (SP) was calculated according to the following ratio: SP = sucrose intake (g)/sucrose intake (g) + water intake (g).

### 3. Western Immunoblotting

After being anesthetized with sodium pentobarbital (40 mg/kg) intraperitoneally, DG tissue was extracted under a dissection microscope (Zeiss, Germany), washed three times with cold PBS, and homogenized in ice-cold lysis buffer (containing 20 mmol/L Tris, pH 7.5, 150 mmol/L NaCl, 1 mmol/L EDTA, 1 mmol/L EGTA, 1% Triton X-100, 2.5 mmol/L Na-Pyrophosphate, 1 mmol/L b-glycerophosphate, 1 mmol/L Na3VO4, 1 g/mL leupeptin and 1 mmol/L PMSF). Samples were centrifuged at 14,000 rpm for 15 min. Supernatant protein concentration determined with a spectrophotometer (Thermo). After loading buffer was added to samples, they were boiled for 3 min. Protein samples (50 µg) were electrophoresed on 10% Bis–Tris gels (Invitrogen) and transferred to PVDF membranes. Membranes were blocked with Tris-buffered saline (TBS) containing 1% bovine serum albumin, 5% nonfat milk powder (w/v). Membranes were incubated overnight with a rat monoclonal antibody (BD) for bcl-2 (1∶200) and bax (1∶500) at a temperature of 4°C. Membranes were washed 3 times with TBS containing 0.05% Tween 20 (TBST) for 15 min and incubated with a second antibody [anti-guinea pig IgG (Santa Cruz), 1∶1000 in TBST] for 2 h. Blots were washed 3 times with TBST for 15 min. Signal intensities for bcl-2 and bax were normalized against the internal GAPDH control. Protein expression levels of bcl-2, bax and β-action protein were quantified by densitometry (TotalLab, version 1.1, UK) for each blot. The density ratio for the bands was calculated. All data are expressed relative to control as follows: relative density (%) = density (study groups)/density (control group) × 100. Resulting measurements represent the relative expression of apoptotic proteins.

### 4. Real-time RT-PCR

Total RNA was extracted from the dentate gyrus using Trizol reagent kit (Invitrogen). Total RNA (1 µg) was used as a template for first strand cDNA synthesis with random primers in the Promega RT System. All the PCR primers were designed by Primer Premier 5.0 (Table 1). One milliliter of template was mixed into Master Mix (10× SYBR Green PCR buffer, 25 mM MgCl2, 2.5 mM dNTP, 10000× Sybr green, Taq DNA polymerase) plus each primer (20 µM). The reaction mixture was brought up to a final volume of 25 µL with RNase-free deionized water. Amplification conditions were 2 min at 30°C, 10 min at 95°C (Taq DNA polymerase activation), followed by 40 cycles of 20 s at 94°C (denaturing), 20 s at 55°C (annealing) and 30 s at 72°C (extension). Real-time RT-PCR was performed by monitoring the increase in fluorescence intensity of the SYBR Green dye with a Rotor-Gene 3000 Real-time PCR apparatus (Corbett Research) according to the manufacturer’s instructions. All measurements were performed in triplicate. Real-time RT-PCR data were represented as Ct values, where Ct was defined as the threshold cycle of PCR when amplified product was first detected. To minimize intra- and inter-assay variability caused by differences in PCR efficiency, the quantity of 5-tissue. The Ct or threshold value of the target sequence is directly proportional to the absolute concentration when compared with the threshold value for reference genes. The relative expression level of bcl-2 and bax were plotted as fold change compared to control and determined by the 2- ΔΔ Ct method [21], a relative quantification algorithm. The factor X by which the amount of the changed gene can be calculated with the formula: X = 2-ΔΔ Ct. where ΔΔCt = (Ct, bcl-2 or bax-Ct, β-actin) control- (Ct, bcl-2 or bax -Ct, β-actin) sample.

**Table 1:** The primers for real-time PCR.

Gene	Primers
bcl-2	5′-CTGGTGGACAACATCGCTCTG-3′ sense
	5′-GGTCTGCTGACCTCACTTGTG-3′ antisense
bax	5′-TCCAGGATCGAGCAGA-3′ sense
	5′-AAGTAGAAGAGGGCAACC-3′ antisense
synaptophysin	5′- CCCTACATTCACCCACTTCTCC -3′ sense
	5′- TTATCTCCTCTCTGCCCGTTTC -3′antisense
β-actin	5′-ATTGTAACCAACTGGGACG-3′ sense
	5′-TTGCCGATAGTGATGACCT-3′ antisense

### 5. TUNEL Staining and Data Analysis

After being anesthetized with sodium pentobarbital (40 mg/kg) intraperitoneally, the rats were transcardially perfused with a left ventricular cannula with 100 mL of normal saline solution, which was followed by 200 mL of freshly prepared 4% phosphate-buffered paraformaldehyde (pH 7.4). Terminal deoxynucleotidyl transferase-mediated dUTP-biotin nick end labeling (TUNEL) staining was performed with the In Situ Cell Death Detection Kit, POD (Roche Applied Science, Mannheim, Germany). Twelve-micrometer-thick frozen coronal sections underwent the following protocol: Slides were heated at 60°C followed by xylene wash, a graded series of ethanol washes and double distilled water washes. Slides were incubated with protease K incubation for 15–30 min at 21°C–37°C followed by 1% Triton X-100 washing for 10 min. Slides were rinsed with 0.1 M PBS, incubated with 50 µL of TUNEL reaction mixture for 1 hour at 37°C and followed by another incubation for 30 min at 37°C with 50 µL of converter-peroxidase. After 3 washes in 0.1 M PBS for 5 minutes, sections were incubated for 5 min at room temperature with 50 µL of DAB substrate solution and then rinsed again with 0.1 M PBS. After these treatments, the slides were analyzed with light microscopy at 400× magnification (BX51; Olympus, Tokyo, Japan). Coded slides were initially examined for the presence TUNEL-positive cells in the dentate gyrus (including SGZ and hilar) subfields of the hippocampus by an examiner blind to the group assignment of each animal. The number of TUNEL-positive neurons and total neurons in the dentate gyrus region was counted in 3 different fields for each section at 200 magnification. The total number of TUNEL-positive neurons and total neurons found in the 10 selected sections of the hippocampus was calculated, and the mean number of cells per section was used for comparison. The extent of apoptosis was also calculated and expressed as a ratio of TUNEL-positive neurons versus total neurons.

### 6. Statistical Analysis

Values are presented as means ± Standard Deviation (S.D). Sucrose tests along with locomotor and rearing activity were analyzed by repeated measurement ANOVA with treatment (control, MCAO, CMS, MCAO+ CMS, DAPT) and day (baseline, day 28) as the two factors. The quantity of TUNEL-positive neurons as well as ratios of protein and gene expression was compared among groups by one-way analysis of variance. One-way or two-way ANOVA was supported by the Bonferroni post hoc tests for multiple comparisons. *P*<0.05 was considered significant.

## Results

### 1. Behavioral Tests

Table 2 shows open-field activities and relative sucrose intake at baseline and on day 28 after ischemia. Results from two-way ANOVA indicated that MCAO+CMS significantly reduced the locomotor activity [F (4, 50) = 12.55, *P*<0.0001] and the frequency of rearing [F (4, 50) = 14.67, *P*<0.0001]. Bonferroni post hoc tests confirmed that the locomotor and rearing activities of the MCAO+CMS group animals were significantly lowered (*P*<0.001 in all cases; in comparison to the baseline value and the CON group level) on day 28 after ischemia. DAPT significantly increased the frequency of rearing (*P*<0.01) but did not significantly increase the locomotor activity (*P*>0.05) in the MCAO+CMS animals.

Two-way ANOVA performed on sucrose intake indicated a significant effect in MCAO+CMS [F (4, 50) = 19.23, *P*<0.0001]. Bonferroni post hoc tests confirmed that sucrose intake in the MCAO+CMS group animals was significantly reduced (*P*<0.001 in comparison to the baseline value and the CON group level) on day 28 after ischemia. DAPT significantly increased the sucrose intake in the MCAO+CMS animals (*P*<0.01).

**Table 2:** Open Field Activities and Relative Sucrose Intake (%) of All Groups at Baseline and at 28 Days after Ischemia.

Group(n)	Locomotor	Activity	Rearing	Activity	Sucrose	preference (%)
	Baseline	Day28 ISCH	Baseline	Day28 ISCH	Baseline	Day28 ISCH
CON(6)	51.9±2.1	45.9±2.3	18.4±1.3	16.5±0.8	72.7±2.5	71.3±2.0
CMS(6)	52.1±1.8	40.1±2.0^a^	17.8±0.8	11.2±0.7^a^	71.7±2.6	62.1±3.1^a^
ISCH (6)	50.9±1.7	42.1±2.1^b^	18.1±1.0	12.1±0.6^a^	71.8±2.0	68.9±2.0
MCAO+CMS(6)	51.1±2.0	35.6±1.6^a^	18.0±1.4	9.4±0.7^a^	71.5±3.1	52.6±1.8^a^
MCAO+CMS+D(6)	51.9±2.0	36.5±1.4	18.4±1.8	11.8±1.5^c^	71.8±2.7	63.4±2.1^c^

Locomotor activity was defined as at least three paws in a quadrant and rearing behaviour defined as the animal standing upright on its hind legs.

The sucrose preference was calculated according to the following ratio: SP  =  sucrose intake (g)/sucrose intake (g) + water intake (g).

Values are means±S.D. See text for the results of relevant statistical tests. CON, control; CMS, chronic mild stress; ISCH, ischemic stroke; MCAO, middle cerebral artery occlusion; D, DAPT(N-[N-(3,5-difluorophenacetyl-L-alanyl)]-S-phenyl-glycine t-butyl ester).

a *P*<0.001CMS, ISCH and MCAO+CMS vs. their respective CON value.

b P<0.05 ISCH vs. their respective CON value.

c P<0.01 MCAO+CMS +DAPT vs. MCAO+CMS value.

### 2. TUNEL Staining

**Figure 1:**
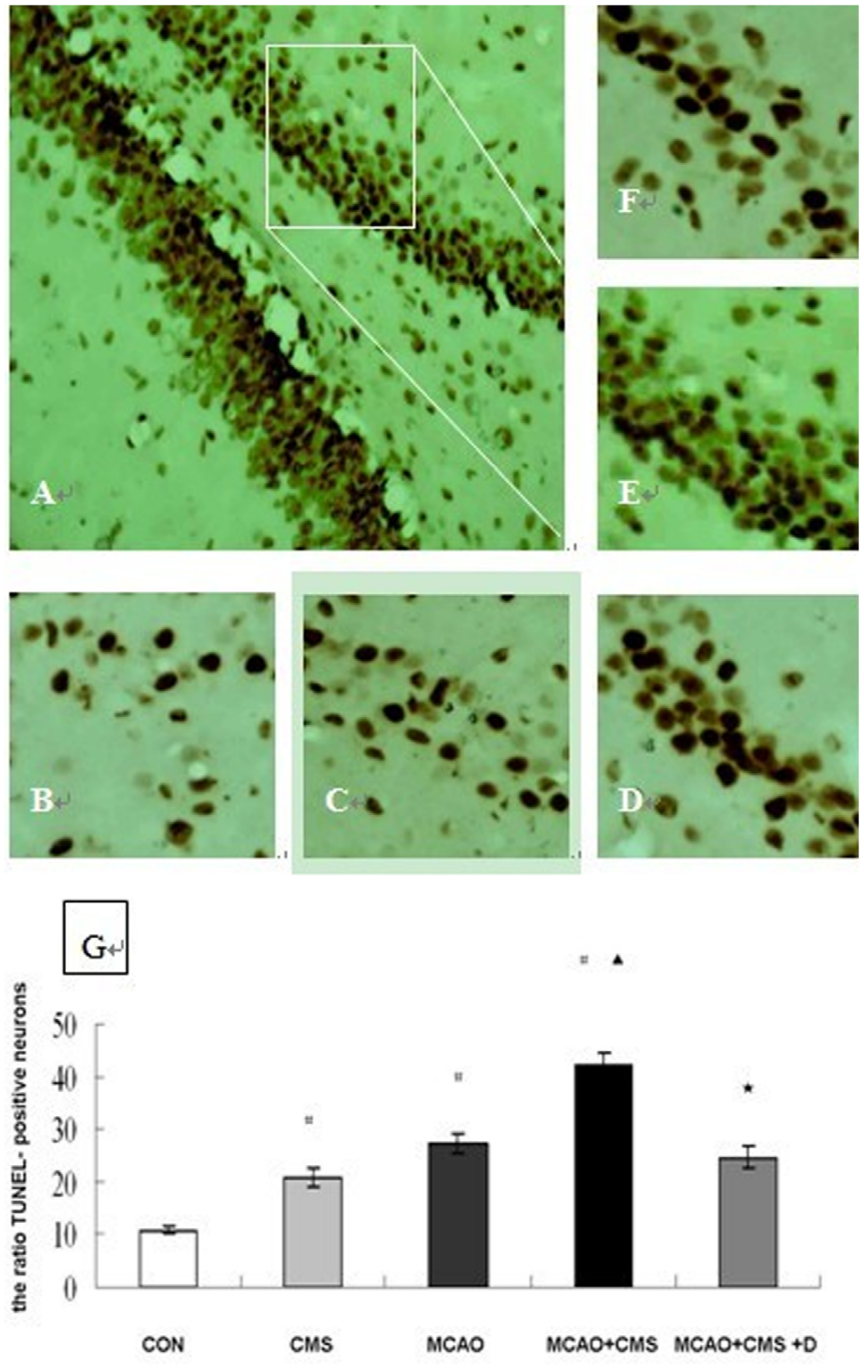
Chronic mild stress increases the number of hippocampal dentate gyrus TUNEL-positive neurons after ischemia in rat. Representative microphotographs of TUNEL-stained hippocampal dentate gyrus at 28 days after ischemia in a rat. A, MCAO+CMS; B, CON; C, CMS; D, MCAO; E, MCAO+CMS; F, MCAO+CMS+D. G, the ratio of TUNEL-positive neurons were analyzed at 28 days after cerebral ischemia, number of each group = 6. ^#^ *P*<0.05 compared with CON, ^▴^ *P*<0.05 MCAO + CMS compared with MCAO, ^★^ *P*<0.05 MCAO + CMS + D compared with MCAO + CMS. CON, Control; CMS, Chronic mild stress; MCAO, Middle cerebral artery occlusion; D, DAPT (N-[N-(3,5-difluorophenacetyl-L-alanyl)]-S-phenyl-glycine t-butyl ester); TUNEL, Terminal deoxynucleotidyl transferase-mediated dUTP-biotin nick end labeling (A, magnification, 20×; B–F, magnification, 40×).

TUNEL staining results are displayed in Figure 1. The ratio of TUNEL-positive neurons in the dentate gyrus region was significantly increased at Day 28 after ischemic injury in the CMS, MCAO and MCAO+CMS group (20.8±1.7%, 27.4±1.9%, 42.1±2.2% vs. 10.8±0.6%, all *P*<0.05). At the same observation time, CMS increased the ratio of TUNEL-positive neurons in ischemia rats (42.1±2.2% vs. 27.4±1.9%, *P*<0.05), and DAPT decreased the number of TUNEL-positive neurons in the rats of the MCAO+CMS group (24.6±2.1% vs. 42.1±2.2%, *P*<0.05).

### 3. Apoptosis-related Gene Bax and Bcl-2 Immunoblots

**Figure 2:**
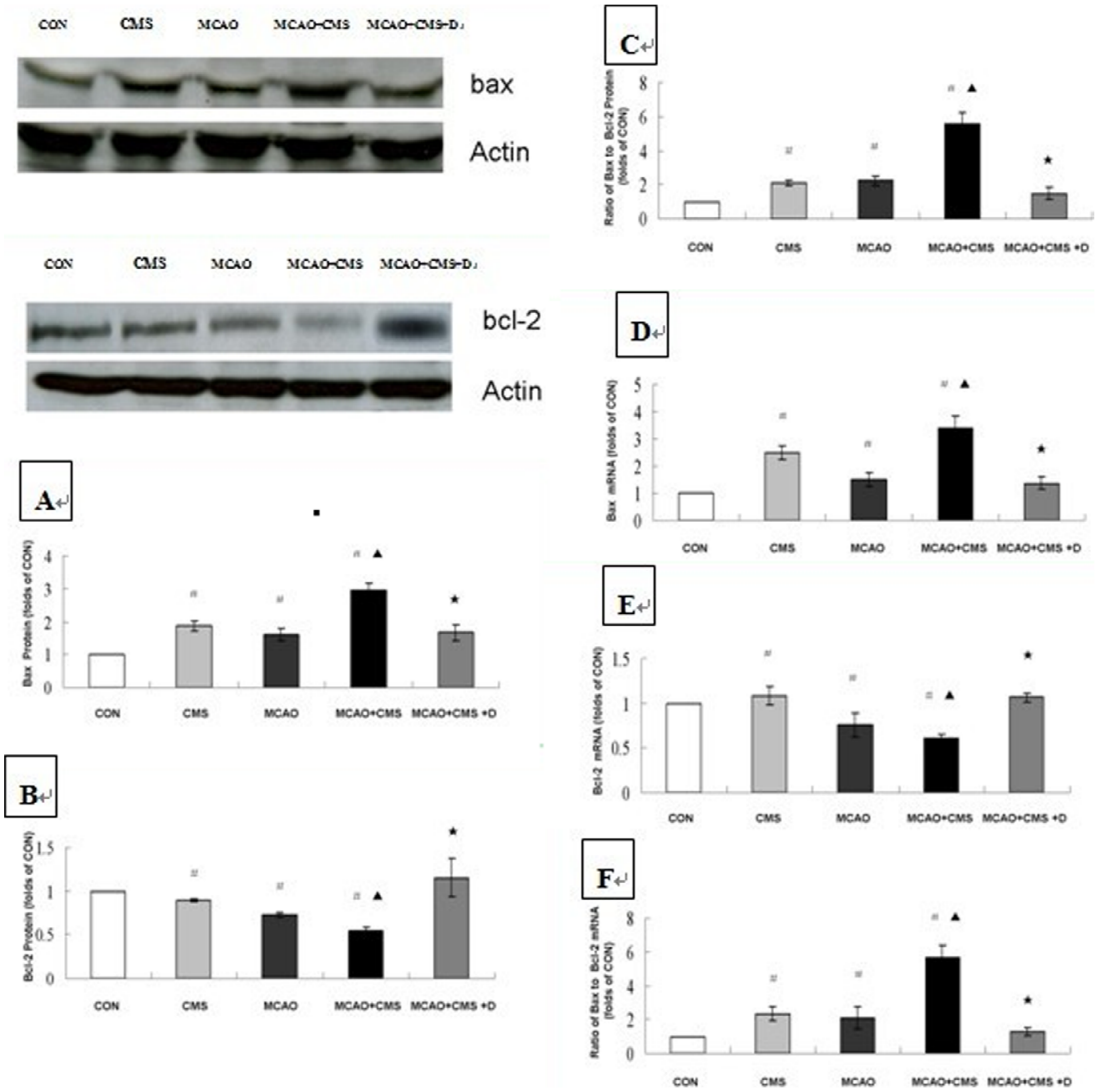
Effects of a gamma-secretase inhibitor on apoptosis-related genes Bax and Bcl-2 in the dentate gyrus of adult ischemic rats in response to chronic mild stress. Western blotting analysis with anti-Bax and anti-Bcl-2 was performed to quantify protein levels of Bax (A), Bcl-2 (B) and ratio of Bax to Bcl-2 (C) in response to chronic mild stress and the effects of gamma-secretase inhibitor DAPT on Day 28 after ischemia surgery. Real-time PCR was also performed to investigate gene expression of Bax (D), Bcl-2 (E) and ratio of Bax to Bcl-2 (F) in response to chronic mild stress and the effects of gamma-secretase inhibitor DAPT on Day 28 after ischemia surgery, number of each group = 6. Representative photographs from three to four independent experiments are shown. Statistically significant differences between CMS, MCAO and MCAO+CMS compared with CON are indicated (^#^ *P*<0.05), MCAO+CMS compared with MCAO are indicated (^▴^ *P*<0.05), or MCAO+CMS+D compared with MCAO+CMS are indicated (^★^ *P*<0.05), as determined by Bonferroni correction. CON, Control; CMS, Chronic mild stress; MCAO, Middle cerebral artery occlusion; D, DAPT (N-[N-(3,5-difluorophenacetyl-L-alanyl)]-S-phenyl-glycine t-butyl ester).

The mean Bax/Bcl-2 ratio was higher in the CMS, MCAO and MCAO+CMS groups relative to the CON group (*P*<0.05), The mean Bax/Bcl-2 ratio was higher in MCAO+CMS group compared to the MCAO group (*P*<0.05). DAPT decreased the Bax/Bcl-2 ratio in the MCAO+CMS group. A substantial difference in Bax/Bcl-2 ratio between the individual groups resulted from differences in the Bax and Bcl-2 levels. Individually, the mean Bax level was higher in the CMS, MCAO and MCAO+CMS groups relative to the CON group (*P*<0.05). CMS increased Bax levels in ischemic rats, which was reversed by DAPT administration. Additionally, compared with the CON group, the mean Bcl-2 level decreased in the CMS, MCAO and MCAO+CMS groups (*P*<0.05). CMS reduced Bcl-2 gene expression in ischemia-treated rats, which was reversed by DAPT treatment. See Figure 2 A–C.

### 4. Apoptosis-related Bax and Bcl-2 Real-time PCR

Mean Bax gene expression was higher in the CMS, MCAO and MCAO+CMS groups relative to the CON group (*P*<0.05). CMS induced Bax gene expression in ischemia-treated rats. This effect was reversed by DAPT administration. Compared with the CON group, mean Bcl-2 gene expression decreased in the CMS, MCAO and MCAO+CMS groups (*P*<0.05). CMS decreased Bcl-2 gene expression in the ischemia rats, which was reversed by DAPT treatment. See Figure 2 D–F.

### 5. Synaptic Density

**Figure 3:**
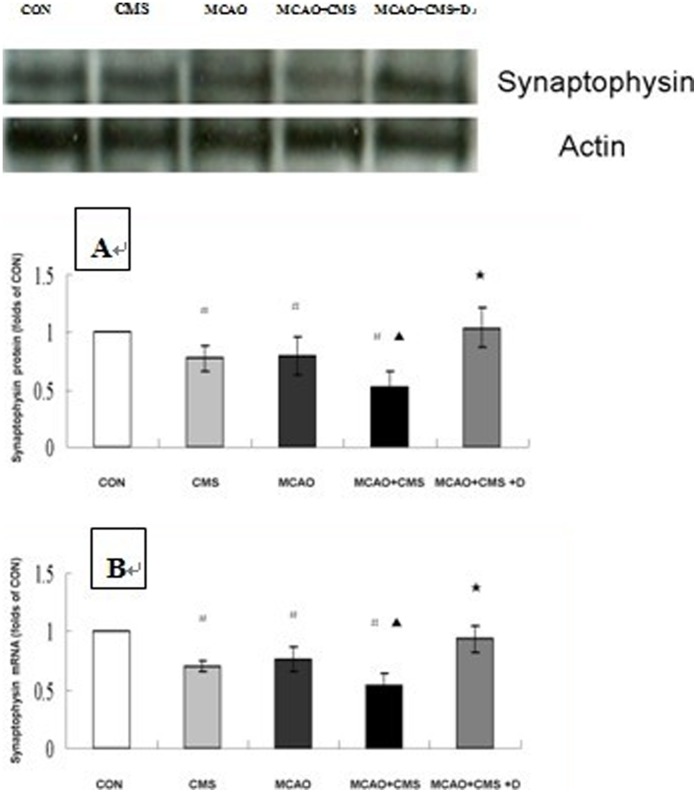
Synapse-related gene synaptophysin in the dentate gyrus of adult ischemic rats in response to chronic mild stress and the effects of gamma-secretase inhibitor. Western blotting analysis using anti-synaptophysin was performed to investigate protein levels of synaptophysin (A) following chronic mild stress and the effects of gamma-secretase inhibitor DAPT on Day 28 after ischemia surgery. Real-time PCR was also performed to quantify gene expression of synaptophysin (B) in response to chronic mild stress and the effects of gamma-secretase inhibitor DAPT on Day 28 after ischemia surgery, number of each group = 6. Representative photographs from three to four independent experiments are shown. Statistically significant differences CMS, MCAO and MCAO+CMS compared with CON are indicated (^#^ *P*<0.05), MCAO+CMS compared with MCAO are indicated (^▴^ *P*<0.05), or MCAO+CMS+D compared with MCAO+CMS are indicated (^★^ *P*<0.05), as determined by Bonferroni correction. CON, Control; CMS, Chronic mild stress; MCAO, Middle cerebral artery occlusion; D, DAPT (N-[N-(3,5-difluorophenacetyl-L-alanyl)]-S-phenyl-glycine t-butyl ester).

Figure 3 A shows western blots and the relative density ratio of the bands in all groups at Day 28 after ischemic surgery. Protein levels of synaptophysin in dentate gyrus were significantly deflated in the stressed, ischemic and MCAO+CMS individuals compared to the controls (*P*<0.05). Furthermore, there was a decrease in synaptophysin protein expression in the MCAO+CMS animals compared to the ischemia rats (*P*<0.05). DAPT significantly stimulated synaptophysin protein expression in the PSD treated animals (*P*<0.05).

Compared with controls, the gene expression of synaptophysin in the stressed, ischemia and MCAO+CMS animals decreased significantly at Day 28 (*P*<0.05). The gene expression in MCAO+CMS animals decreased significantly compared with ischemia subjects at Day 28 (*P*<0.05). DAPT significantly stimulated higher gene expression of synaptophysin in the CMS-treated ischemia animals (*P*<0.05). See Figure 3 B.

## Discussion

This study examined neuronal cell death, apoptosis-related markers (pro-apoptotic Bax and anti-apoptotic Bcl-2) and synaptic density in the hippocampal dentate gyrus of adult ischemic rats in response to CMS. Concurrently, the effects of the gamma-secretase inhibitor DPAT were assessed. Adult rats were exposed to the CMS paradigm after left middle cerebral artery occlusion (MCAO). Open-field and sucrose consumption testing were employed to assay depression-like behavior. Expression of apoptosis-related genes and the synaptic density-related gene synaptophysin were measured on Day 28 after surgery. The most striking finding in the current study is that CMS induced depressive behaviors in ischemic rats, which was accompanied by elevated ratios of Bax/bcl-2, TUNEL staining neuron and reduced expression of synaptophysin in the dentate gyrus. These effects were reversed by gamma secretase inhibitor DAPT administration.

In agreement with our previous findings [20], the current MCAO+CMS protocol induced behavioral changes in ischemic animals, including reduced sucrose preference and decreased activity. Reduced sucrose preference indicates desensitization of the brain reward mechanism (anhedonia), which is consistent with the behavioral correlates of depressive-like symptoms in post-stroke humans. Although the detailed etiology of post-stroke depression remains unclear, it appears to be multifactorial rather than “purely” biological or psychosocial in origin. Thus, psychological and social stressors associated with stroke are considered to be the primary cause of depression [20], [22], [23].

Additionally, the hippocampus is an important brain structure involved in depressive disorders. We focused on hippocampal responses to chronic mild stress, including neurogenesis in the dentate gyrus, which has been associated with depressive disorders. Ischemia-stimulated neurogenesis was impaired in ischemic stroke rats experiencing CMS, which suggests that ischemia-induced neurogenesis might be vital in post-stroke cognitive recovery. Nevertheless, neurogenesis and depressive symptoms were seemingly inconsistent between the MCAO+CMS animals and controls because the MCAO+CMS animals display higher levels of neuronal differentiation and a depression-like syndrome. These findings highlight the importance for newborn neurons to perform their physiological function by involving themselves in neural circuits.

Our current findings resolve the previously mentioned inconsistency. Neuronal cell death in the hippocampus might occur via apoptosis, a consequence of ischemia [17], [18], as we observed apoptotic injury in the dentate gyrus of ischemic rats. Previous research demonstrated that ischemia-induced neuronal death occurs via apoptosis as well as necrosis [24]–[30]. Unlike the acute damage of necrosis, apoptotic injury potentially occurs in milder forms of ischemic damage and requires time to develop [28], [31], [32].

Approximately a month after a stroke, CMS aggravated the apoptotic injury in the ischemia animals. During this period of time, neural stem cells proliferate, migrate and differentiate into neurons. CMS-induced apoptotic injury is potentially responsible for the formation of new neurons and their integration into preexisting neural circuits. It was reported that newborn cells integrate into the granule cell layer 4–10 days after their birth. They extend axons into the CA3 region and begin receiving synaptic contacts, which suggests that they make synapses long before they are fully mature [33]–[37]. It takes 4 months after their generation for these cells to obtain a mature morphology (e.g. soma size, total dendritic length, dendritic branching and spine density) (513). Before they are mature, neurons created in the DG must be established synaptically into the circuitry by 4–8 weeks after their birth [38]. In contrast, newborn cells that remain on the interface of the hilus exhibit electrophysiological properties that are characteristic of immature neurons (paired-pulse facilitation, lower threshold for induction of long-term potentiation (LTP), and robust LTP) [39].

Augmentation of ischemia-related neurogenesis was regarded as a potential target for the amelioration of post-stroke depressive symptoms. Previous evidence from our group demonstrated that improved proliferation and neuronal differentiation in newborn cells were mediated by antidepressant action and alleviated symptoms [12]. It is critical for the newborn cells to form synapses with older neurons. In ischemia animals, CMS-promoted apoptotic injury potentially contributes to impairment of circuit integration in mature newborn cells. However, we could not ascertain whether both preexisting cells and newborn cells were affected. These findings provide a possible explanation for inconsistencies highlighted in our previous study. In summation, it is very important for newborn cells to form synapses with older cells. These two cell types might be affected by CMS-promoted apoptosis injury, which has been regarded as an etiological factor in post-stroke depression and potential drug target.

In our previous study, we found that Notch signaling fulfills important roles in ischemia-stimulated neurogenesis [13]. In this study, elevation of the Bax/bcl-2 ratio, neuronal TUNEL staining neuron and synaptophysin expression in the dentate gyrus were reversed by gamma-secretase inhibitor DAPT administration. Our findings indicate that Notch signaling was involved in CMS-related apoptosis injury and synaptic integration of newborn cells in the ischemic stroke animals. The mechanism requires further elucidation.

The interpretation of the data presented in this study has certain limitations. First, we found that CMS promoted apoptosis injury in the dentate gyrus, but we cannot confirm whether the loss of newborn or preexisting cells occurs in response to CMS after stroke. Second, the CMS combined with social isolation regimen used in the current study attempt to model the clinical situation of the PSD patients. However, the patients will not encounter severe environmental stress, like food and water deprivation, disturbance of day and night rhythm or temperature shocks, and they still has social contacts, e.g., medical personnel, also visits by relatives. Furthermore, we can not exclude the above-mentioned stressors might directly affect physiological functions besides causing stress, possibly directly altering gene expression or behaviour. Finally, CMS protocol is widely used for depression research. Post-stroke depression is considered as a complex syndrome [40], and the CMS protocol seems to be not fully representative of a combination of biological, psychosocial and environmental factors present in human PSD, for the observed effect is specific to a combination of two independent brain harming events, being chronic quite severe stress and ischemic injury.

### Conclusions

In spite of these limitations, our results raise the possibility that post-stroke CMS was involved in deficits of hippocampal newborn cells by not only inducing apoptosis, but also impairing the establishment of novel circuits, all these ameliorated by gamma secretase inhibitor DAPT. On the other hand, PSD is considered as a complex syndrome. The current CMS protocol PSD model induced reduction in sucrose intake that might be regarded as behavioural correlates of depressive-like symptoms in post-stroke humans, indicating that depressive syndrome consequence of chronic stress. We noticed that MCAO alone also resulted in suppression of sucrose intake, MCAO+CMS induced depressive-like symptoms seems to be regarded as synthestic effects of MCAO and CMS. Although DAPT treatment could bring the sucrose intake back to levels of CMS alone, but still didn’t reach the values of MCAO alone, our findings indicated that post-stroke depressive syndrome therapeutically benefit from blocking gamma secretase mediated Notch signaling, and whether this signaling pathway could be therapeutic target need to be further investigated.
